# A Video Interview With James M. Stuzin, MD: The SMAS: Past, Present, and Future

**DOI:** 10.1093/asjof/ojac038

**Published:** 2022-05-02

**Authors:** James M Stuzin, Lorne King Rosenfield

**Affiliations:** Department of Plastic Surgery, University of Miami School of Medicine, Miami, FL, USA; Department of Plastic Surgery, University of California San Francisco, San Francisco, CA, USA

This edition of *Second Thoughts on First Thoughts* brings us another icon in aesthetic surgery, James M. Stuzin, MD. Our conversation focused on his first love in aesthetic surgery—the superficial muscular aponeurotic system (SMAS). You will be treated to a travelogue along his learning curve: from first endeavors to better understand facial anatomy with years of painstaking anatomical study, through its successful application in his operating room, to today, where he is still tinkering—again in both the laboratory and operating room—with strategies to achieve ever better aesthetic contouring of the jaw.

Dr Stuzin epitomizes the kind of surgeon that Halsted originally challenged his own colleagues to become: not just a technician but also a “surgeon-scientist.” As Dr Stuzin urges, by returning to the anatomy laboratory, to your surgical texts, and to your own cases, the subsequently earned confidence in facial anatomy will pay huge dividends in your operating room. So, please take a minute to enjoy this short but fruitful conversation that will surely inspire you as well (Video).

